# Accumulation of Cardiovascular and Diabetes Medication among Apparently Healthy Statin Initiators

**DOI:** 10.1371/journal.pone.0117182

**Published:** 2015-02-06

**Authors:** Piia Lavikainen, Maarit Jaana Korhonen, Risto Huupponen, Arja Helin-Salmivaara

**Affiliations:** 1 Department of Pharmacology, Drug Development and Therapeutics, University of Turku, Turku, Finland; 2 Drug Research Doctoral Programme, University of Turku, Turku, Finland; 3 Department of Public Health, University of Turku, Turku, Finland; 4 Department of Clinical Pharmacology, Tykslab, Turku University Hospital, Turku, Finland; 5 Unit of Primary Health Care, Hospital District of Helsinki and Uusimaa, Helsinki, Finland; University of Catania, ITALY

## Abstract

**Purpose:**

To characterize accumulation of drug-modifiable cardiovascular (CV) risk factors in statin initiators who had no prior medication or hospitalizations for CV disease or diabetes.

**Methods:**

A cohort of 45-75-year-old statin initiators in Finland with no prior CV diseases, diabetes or medication for these conditions was followed up for 24 months after statin initiation for accumulation of CV and diabetes drugs. The number of drugs was measured semi-annually since the first statin purchase and analyzed by growth mixture modeling.

**Results:**

Of the 11 948 apparently healthy statin initiators, 34% purchased at least one additional CV or diabetes drug during the subsequent 24 months. Of those, 20% redeemed no other CV or diabetes drugs at statin initiation but started to accumulate them after 18 months of follow-up, receiving on average 1.3 other drugs at 24 months. The majority, 59%, redeemed on average 0.5 drugs at statin initiation and accumulated 1.5 drugs by the end of 24-month follow-up. Seventeen percent received 1 additional drug at statin initiation, accumulating on average 3 drugs. The remaining 4% with an average of 2 CV or diabetes drugs at statin initiation redeemed 3.5 additional drugs during the follow-up.

**Conclusions:**

Two years after statin initiation, 2 in 3 apparently healthy initiators could still be defined as such as reflected by CV and diabetes medication use.

## Introduction

During the last 15 years, recommendations and indications for statin therapy have broadened from secondary prevention (prevention of progression of the disease and prevention of recurrent events in patients with existing cardiovascular [CV] disease) [1–3] to primary prevention of CV disease in individuals with CV risk factors [4,5]. Implementation of the current recommendations is likely to place a significant burden on societies and insurers. For example, following the criteria of the 2013 guideline [5] from the American College of Cardiology (ACC) and the American Heart Association (AHA) could result in about 1 in 3 American adults (of whom ~33 million at ≥7.5% and ~12 million at 5.0–7.4% 10-year risk of an atherosclerotic CV event) being recommended for consideration of statin therapy [6]. Even in the era of generic statins, however, statin therapy in low-risk populations may not be universally cost-effective [7–9].

In population-based studies that have classified statin initiators by their estimated CV disease risk level, a shift towards low-risk individuals has been found, including those with no CV disease, diabetes, or prior medication for these conditions. In British Columbia, Canada, 17% of statin initiators in 1996–2001 had no marker of previous CV disease in the hospital discharge register or in the prescription register [10]. In Denmark, the proportion of statin initiators with no prior CV disease, diabetes, or medication for these conditions increased from 20% to 26% between 1996 and 2009 [11]. A similar shift, from 24% to 28%, was observed in Finland between 1999 and 2008 [12].

In the population-based studies, initiators defined as being at low CV disease risk at the time of statin initiation may, however, be misclassified as such if the definition is based on register data on the period prior to or on the date of the initiation. Various CV risk factors may be clinically present at the time of statin initiation but not detectable in health care registers until later as they can be targeted one at a time. In this study, the aim was to characterize accumulation of CV and diabetes medication as indicators of multiple CV risk factors among apparently healthy statin initiators defined as individuals aged 45–75 years who had no prior medication or hospitalizations for CV disease or diabetes. We applied growth mixture modeling to gain understanding about accumulation of CV and diabetes medication during a 2-year follow-up. To our knowledge, this kind of information has not been published previously.

## Materials and Methods

### Data sources

We used data from administrative databases generated through the universal health care and drug reimbursement systems covering the 5.4 million residents of Finland. We identified prescription records with the Prescription Register run since 1994 and managed by the Social Insurance Institution (SII) [13]. This register contains records of prescription drug purchases reimbursed to residents in non-institutional settings. For each drug, the data include the dispensing date, the Anatomical Therapeutic Chemical (ATC) classification code [14], and the quantity dispensed. Patients staying at public nursing homes or hospitals without interruption for over 90 days are not eligible for drug reimbursement, and their purchases are not registered. These long-term institutionalized patients can be identified by a separate SII register.

We identified hospitalizations from the Finnish Care Register, managed by the National Institute for Health and Welfare. The register, covering all Finnish hospitals, includes individual administrative data on main and additional discharge diagnoses, and the admission and discharge dates. The 10th revision of the International Classification of Diseases (ICD-10) has been in use since Jan 1, 1996. The data from the above databases were linked anonymously using encrypted personal identifiers.

### Design

A cohort of initiators of simvastatin (ATC code C10AA01), lovastatin (C10AA02), pravastatin (C10AA03), fluvastatin (C10AA04), atorvastatin (C10AA05), or rosuvastatin (C10AA07) therapy between Apr 1 and Dec 31, 2006 and aged 45–75 years was followed up for 730 days (24 months) for accumulation of CV or diabetes medication. The initiation was defined as not having purchased any statin (C10AA01–C10AA07, C10BA02) since Jan 1, 1994. In addition, we excluded those who had purchased CV medication (antithrombotic agents [ATC class B01], cardiac glycosides [C01A], antiarrhythmic drugs [C01B], nitrates [C01DA], centrally acting antihypertensive drugs [C02], diuretics [C03], peripheral vasodilators [C04], beta blockers [C07], calcium channel blockers [C08], angiotensin-converting enzyme [ACE] inhibitors [C09A, C09B], or angiotensin receptor blockers [ARB] [C09C, C09D]) or diabetes drugs (A10) during 3 years preceding statin initiation. Furthermore, those hospitalized for diabetes (ICD-10 codes E10–E14), hypertension (I10–I14), atherosclerotic CV disease (I20–I25, I63, I69.3, I70, I71), cardiac insufficiency (I50), or for coronary revascularization procedures in the preceding 7 years and those hospitalized for atrial fibrillation (I48) during the preceding year were excluded. In addition, individuals living in institutions within 3 years prior to the initiation were excluded. The outcome of interest was semi-annually measured accumulated number of CV and diabetes drugs as listed above, a surrogate of CV risk factors. The follow-up started on the date of the first statin purchase and ended in death, long-term institutionalization, or after 730 days (24 months) of follow-up, whichever came first. The vital statistics came from the SII. We described the latent classes (see below) according to age at statin initiation, sex, and hospitalizations for other than CV disease or diabetes diagnoses in the 365 days preceding the initiation.

Adherence to statin therapy during the first year of statin use was measured as the proportion of days covered (PDC); i.e. the number of tablets available to the patient divided by 365 [15]. The use of statin therapy during the second year of follow-up was defined as having a statin purchase during the second year or as having tablets left over from a previous purchase taken place during the first 365 days.

### Statistical analyses

We applied growth mixture modeling to identify unobserved homogeneous subpopulations (latent classes) within a larger heterogeneous population and to group patients according to the similarities of their trajectories on accumulating CV and diabetes drugs over the 24-month follow-up using the Mplus Version 7 program [16]. Growth mixture modeling is based on an assumption that each individual’s count over time results from him/her being a member of a latent, unobserved class while conventional growth models assume that individuals come from a single population and estimate a single growth factor that might lead to an oversimplified estimate of change among individuals with different health status [17–18]. In addition to identifying subpopulations in a *post-hoc* manner, growth mixture models (GMM) describe differences in change between and within the unobserved subpopulations.

The outcome variable, defined as the cumulative number of distinct CV or diabetes drugs (identified by the fifth ATC level, excluding statins), was measured semi-annually since the date of the first statin purchase and it was assumed to be Poisson distributed. When constructing the models, we started by considering linear and quadratic trends over time for the observed outcome variables with 1 to 5 latent classes. We evaluated the appropriateness of the models using Bayesian information criteria (BIC), with the smallest value indicating the best fit, and the Lo-Mendell-Rubin Likelihood Ratio test, with P-values less than 0.05 indicating that a more parsimonious model fits data worse than a model with more parameters [19]. In GMMs, each individual’s probabilities to belong to each of the latent classes are calculated based on how her/his trajectories match the mean trajectory of each of the classes. Probabilities are then used to assign individuals to the class they most likely belong to. Classification accuracy was evaluated using the entropy value ranging between 0 and 1, with larger values indicating better accuracy. No less than 1% of the total count in a class was accepted when determining the number of classes.

To aid convergence, variances of linear and quadratic growth factors were fixed to zero [17]. All the models were fitted using random starts, and the estimates from the final model presented here were replicated by rerunning the model with the seeds that produced the best 2 log-likelihood values. This was done to check that results did not arise from local maxima. Once the best fitting model had been identified, we compared baseline characteristics of the latent classes by one-way analysis of variance and the Chi-Square test. Analyses are valid under missing at random assumption, i.e. missing values were not imputed but, instead, parameters and standard errors were estimated with all available data.

### Ethics Statement

Data were obtained from the databases hosted by the Social Insurance Institution and the National Institute for Health and Welfare, Helsinki, Finland that are not public repositories. The Social Insurance Institution and the National Institute for Health and Welfare approved the study protocol. There was no legal requirement for an ethics committee approval because researchers used only de-identified register data and the individuals in the registers were not contacted. No written consent from patients was required either.

## Results

**Fig 1:**
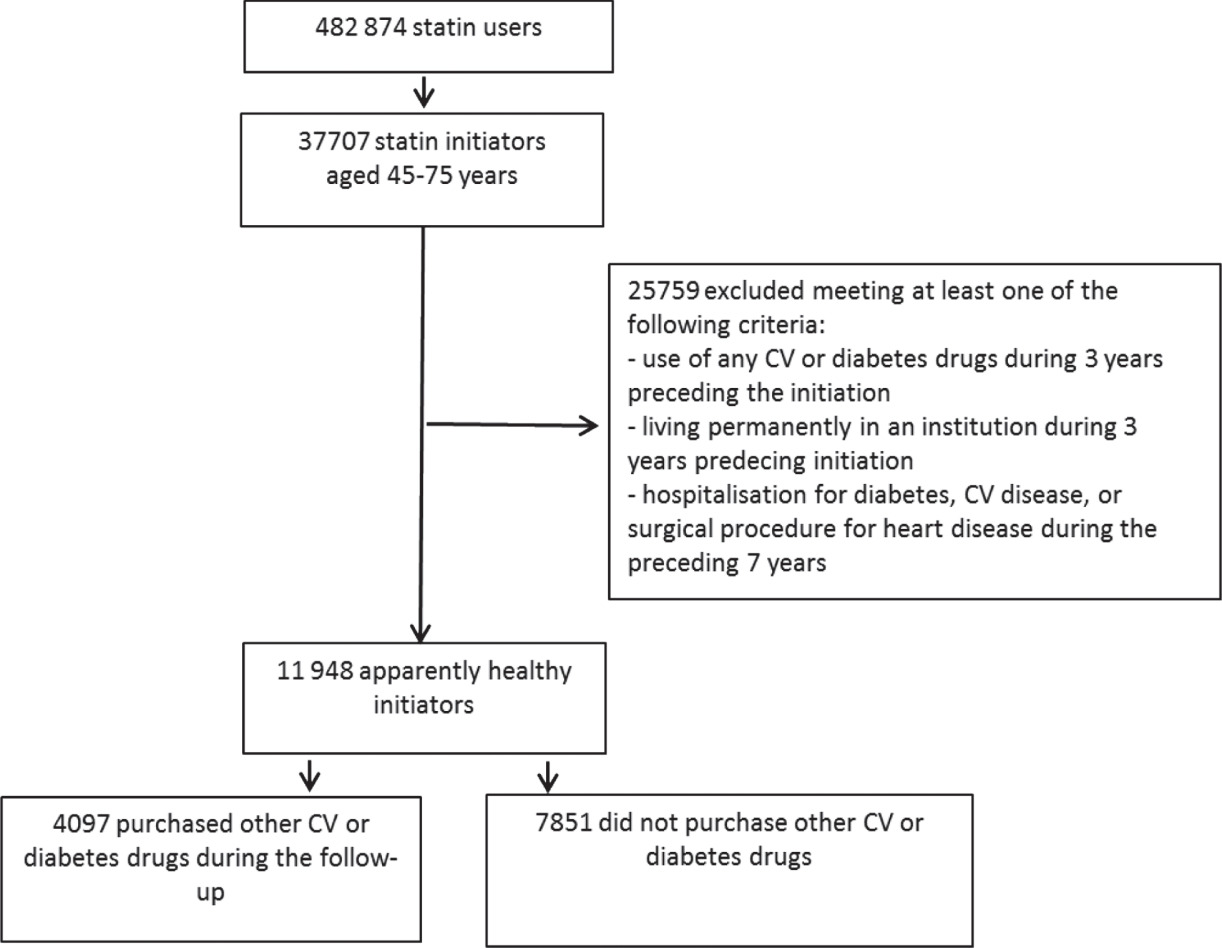
Patient flow between 1^st^ Apr and 31^st^ Dec 2006 with the 24-month follow-up (CV = cardiovascular).

During the last three quarters of 2006, statin therapy was initiated by 37 707 individuals aged 45–75 years (Fig. 1). Of them, 31.7% (11 948) were apparently healthy, i.e., they had no register marker of prior CV disease, diabetes, or medications indicated for these conditions. During the subsequent 24 months since statin initiation, 65.7% (7 851) of these apparently healthy individuals had no purchases of CV or diabetes drugs in addition to statins. Of the remaining 4 097 (34.3%) patients who initiated other CV or diabetes drugs during the follow-up, 42.0% (1 719) initiated another CV or diabetes drug on the day of the statin initiation and altogether 66.1% (2 699) during the first 6 months of the follow-up.

The 4 097 patients who initiated additional drugs were older than those who did not (mean age: 59.3 years [SD = 7.69] versus 58.0 [7.35] years, *p* <0.001). Of women, 31.8% (1 985) and of men, 37.0% (2 112) initiated other CV or diabetes medication (Table 1). The proportion of those who had been hospitalized in the year preceding statin initiation was higher among the initiators than non-initiators, 15.9% vs. 11.8% in women and 17.7% vs. 10.7% in men (Table 1). Of the initiators of other CV or diabetes medication 1.2% (47) and of the non-initiators 0.7% (56) were long-term institutionalized or died during the follow-up.

**Table 1:** Characteristics of the 11 948 apparently healthy* statin initiators stratified by purchase of other cardiovascular or diabetes drugs during the 24-month follow-up.

	Women		Men	
	n = 6 246 (52.3%)		n = 5 702 (47.7%)	
	Purchases of other drugs	Without purchases	Purchases of other drugs	Without purchases
	n = 1 985 (31.8%)	n = 4 261 (68.2%)	n = 2 112 (37.0%)	n = 3 590 (63.0%)
Age group, n (%)				
45–49	158 (8.0)	357 (8.4)	287 (13.6)	677 (18.9)
50–59	785 (39.5)	1 906 (44.7)	945 (44.7)	1 746 (48.6)
60–69	748 (37.7)	1 594 (37.4)	669 (31.7)	927 (25.8)
70–75	294 (14.8)	404 (9.5)	211 (10.0)	240 (6.7)
Age, mean (SD)	60.4 (7.6)	59.2 (7.1)	58.3 (7.6)	56.6 (7.4)
Died during the follow-up, n (%)	6 (0.3)	14 (0.3)	25 (1.0)	28 (0.8)
Long-term institutionalized during the follow-up, n (%)	8 (0.6)	5 (0.1)	8 (0.4)	9 (0.3)
Hospitalized for other than cardiovascular diseases during 365 days prior to statin initiation, n (%)	316 (15.9)	502 (11.8)	374 (17.7)	384 (10.7)

* no record of prior cardiovascular diseases or diabetes in the hospital discharge register within 7 years or medication for these conditions within 3 years

Typically, other CV or diabetes drugs were initiated one at a time (85.5% of all initiated drugs during the follow-up) but it was also common to initiate 2 drugs simultaneously (10.7%). During the 24-month follow-up, 51.4% of the initiators of other drugs purchased ACE inhibitors/ARB inhibitors, 33.4% beta blockers, 17.9% diabetes drugs, and 17.3% nitrates (S1 Table). Of the drugs purchased on the day of statin initiation, beta-blockers, calcium channel blockers, diuretics, centrally acting antihypertensive drugs and ACE inhibitors/ARB accounted for 68.2% (1 482) while nitrates accounted for 12.7% (277) and diabetes drugs for 11.4% (247) (S1 Table). Of the 732 diabetes drug initiations, 52.3% (383) took place during the first 6 months (including the statin initiation date) and 14.8% (108), 17.8% (130) and 15.2% (111) in the three 6-month periods thereafter (S1 Table).

**Fig 2:**
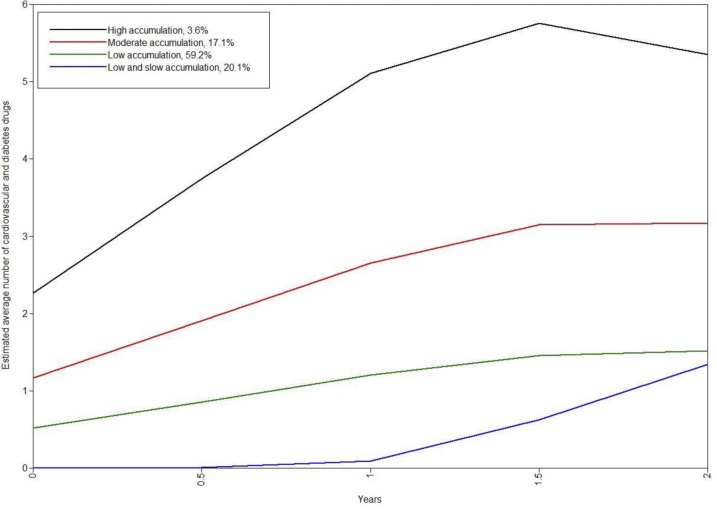
Estimated class trajectories of 4-class quadratic growth mixture models (n = 4 097). Number of other cardiovascular or diabetes drug initiations accumulated during the 24-month follow-up.

GMMs were fitted using data from the 4 097 individuals who initiated at least one other CV or diabetes drug during the 24-month follow-up. Based on the lowest BIC value and estimability, we chose the quadratic 4-class GMM as our final model (Table 2). This model is graphically presented in S1 Fig. Estimated trajectories from the selected 4-class quadratic GMM are presented in Fig. 2. A majority (59.2%) of the individuals who started other CV or diabetes drugs during the follow-up were classified to the “low accumulation” group where patients redeemed typically one other drug during the 6 months after statin initiation. The count remained low until the end of follow-up, being on average 1.5 drugs at 24 months. One fifth of the individuals (20.1%) (the group of “low and slow accumulation”) did not redeem any other CV or diabetes drug simultaneously with the first statin, but started to accumulate them after 18 months, ending up with on average 1.3 CV or diabetes drugs at 24 months. Of the initiators of other drugs, 17.1% were classified to the “moderate accumulation” group that purchased one additional CV or diabetes drug at statin initiation and on average 3 drugs by the end of follow-up. The remaining 3.6% were classified to the “high accumulation” group that redeemed an average of 2 CV or diabetes drugs on the day of statin initiation and a further 3.5 drugs during the follow-up, ending up with an average of 5.5 drugs.

**Table 2:** Estimation results of growth mixture models in the 4097 apparently healthy statin initiators with other drug purchases.

Model	Log-likelihood	Number of parameters	BIC	Entropy	Lo-Mendell-Rubin Likelihood Ratio Test		Proportion of individuals in class				
					2log likelihood	P-value					
Linear GMM							Class 1	Class 2	Class 3	Class 4	Class 5
1-class	-26 136.3	3	52 298	NA	NA	NA	1.000	-	-	-	-
2-class	-25 916.7	6	51 883	0.718	422.27	<0.001	0.200	0.800	-	-	-
3-class	-25 766.4	9	51 608	0.723	289.03	<0.001	0.112	0.196	0.691	-	-
4-class	-25 720.7	12	51 541	0.715	87.91	<0.001	0.600	0.196	0.171	0.033	-
5-class	-25 720.7	15	51 566	0.755	89.28	<0.001	0.600	0.172	0.032	0.196	0.000
Quadratic GMM											
1-class	-25 995.1	4	52 023	NA	NA	NA	1.000	-	-	-	-
2-class	-25 664.1	8	51 395	0.754	642.70	<0.001	0.220	0.780	-	-	-
3-class	-25 511.7	12	51 123	0.749	296.05	<0.001	0.674	0.111	0.215	-	-
4-class	-25 467.2	16	51 068	0.737	82.30	<0.001	0.160	0.215	0.593	0.031	-
5-class	-25 462.5	20	51 091	0.762	9.17	0.001	0.001	0.034	0.165	0.584	0.215

Abbreviations: BIC, Bayesian information criteria;

GMM, growth mixture models;

NA, not available

**Table 3:** Class-specific characteristics of the 4 097 statin initiators with other drug purchases during the 24-month follow-up.

	Low and slow accumulation	Low accumulation	Moderate accumulation	High accumulation	P-value
Posterior probability of patients by class, %^a^	20.1	59.2	17.1	3.6	
Actual number (%) of patients	882 (21.5)	2431 (59.3)	655 (16.0)	129 (3.1)	
Female, n (%)	486 (55.1)	1 216 (50.0)	253 (38.6)	30 (23.3)	<0.001
Age group, n (%)					0.030
45–49	89 (10.1)	270 (11.1)	72 (11.0)	14 (10.9)	
50–59	377 (42.7)	1 044 (42.9)	265 (40.5)	44 (34.1)	
60–69	323 (36.6)	832 (34.2)	214 (32.7)	48 (37.2)	
70–75	93 (10.5)	285 (11.7)	104 (15.9)	23 (17.8)	
Age, mean (SD)	59.2 (7.4)	59.1 (7.70)	59.9 (7.9)	60.6 (8.05)	0.021
Hospitalized in the preceding 365 days, n (%)	114 (12.9)	381 (15.7)	159 (24.3)	36 (27.9)	<0.001
Adherence^b^ during the first 365 days, mean (SD)	0.66 (0.3)	0.69 (0.3)	0.71 (0.3)	0.78 (0.3)	<0.001
No statin purchase since the first 365 days, n (%)	170 (19.3)	529 (21.8)	122 (18.6)	18 (14.0)	0.046
At least one purchase during the follow-up, n (%)					
Diabetes drugs	160 (18.1)	390 (16.0)	152 (23.2)	30 (23.3)	<0.001
Other antithrombotic agents but warfarin	118 (13.4)	285 (11.7)	143 (21.8)	50 (38.8)	<0.001
Warfarin	28 (3.2)	64 (2.6)	51 (7.8)	23 (17.8)	<0.001
Cardiac glycosides and antiarrhythmics	9 (1.0)	20 (0.8)	10 (1.5)	13 (10.1)	<0.001
Organic nitrates	108 (12.2)	254 (10.4)	271 (41.4)	77 (59.7)	<0.001
Centrally acting antihypertensive drugs	3 (0.3)	2 (0.1)	9 (1.4)	7 (5.4)	<0.001
Diuretics	74 (8.4)	215 (8.8)	140 (21.4)	63 (48.8)	<0.001
Beta blockers	235 (26.6)	613 (25.2)	402 (61.4)	118 (91.5)	<0.001
Calcium channel blockers	79 (9.0)	250 (10.3)	145 (22.1)	59 (45.7)	<0.001
ACEI or ARB	327 (37.1)	1229 (50.6)	431 (65.8)	118 (91.5)	<0.001

Abbreviations: CV disease, cardiovascular disease;

ACEI, angiotensin-converting enzyme inhibitor;

ARB, angiotensin receptor blocker

^a^Posterior probability presented in Fig. 2.

^b^Adherence was defined as proportion of days covered.

Of the patients in the “high accumulation” group, 86.0% persisted with statins after the first year of follow-up (Table 3). Corresponding figures were 81.4% for the “moderate accumulation”, 80.7% for the “low and slow accumulation”, and 78.2% for the “slow accumulation” groups. Of the patients not initiating additional drugs, 73.5% continued statin therapy after the first year (not shown in Table 3). Also PDC was the lowest in patients not initiating other drugs (data not shown).

Patients in the “high accumulation” and “moderate accumulation” groups were more often male and older and had more commonly been hospitalized during the year preceding statin initiation than patients in the “low and slow accumulation” or “low accumulation” groups (Table 3).

## Discussion

In Finland in 2006, the majority of apparently healthy statin initiators did not have CV risk factors modifiable by drug therapy as two thirds of the initiators deemed free of atherosclerotic CV disease or diabetes at the initiation did not purchase any other drugs used to treat or prevent these conditions during the subsequent 2 years. About one in 7 (~14%) initiators, however, filled a prescription for at least one other CV or diabetes drug already with their first statin prescription. Overall, four clinically different patterns of accumulating CV and diabetes drugs emerged among those statin initiators who purchased additional medication within the subsequent 2 years.

The estimated patterns of accumulating CV and diabetes drugs differed from each other in terms of number and rate of accumulated drugs. Clinically, the low and slow, and the low accumulation groups were similar, and together they accounted for 81% of the apparently healthy statin initiators (Table 3). In these groups, individuals purchased on average 0–0.5 other CV or diabetes drugs at statin initiation and no more than on average 1.3–1.5 drugs had accumulated by the end of the follow-up. In both groups one in four patients purchased beta blockers, most likely prescribed for either hypertension or coronary heart disease. Nitrates were purchased by 12% of the individuals in the low and slow and by 10% of those in the low accumulation group. Individuals in both of these groups can be assumed to have at least moderately increased CV risk.

The moderate and high accumulation groups differed clinically more clearly from each other (Fig. 2, Table 3). Of the individuals in the moderate accumulation group 61% and 92% of those in the high accumulation group purchased beta blockers (Table 3). Individuals in both groups were obviously at high CV risk already at the time of statin initiation.

There is growing evidence suggesting that statin therapy is associated with an increased risk of new-onset diabetes [20]. In our study population of apparently healthy statin initiators, diabetes was present already at the time of statin initiation in 2% (247). When considering the distribution of all initiations of diabetes drugs (S1 Table), we did not find any signal of an increasing frequency of new-onset diabetes over time. Dietary therapy was not recorded, and we may have missed milder forms of diabetes.

Few findings from other settings are available for comparison with our results. In nearly all studies describing statin users, characteristics of individual patients have been aggregated. However, in defining the apparently healthy, we used the method based on indication hierarchy described by a Danish group [21]. In 2006 in Denmark, approximately a quarter of new statin users aged ≥40 years were apparently healthy [11], which is in line with the 32% in our data.

Vivid debate on the benefits of statin therapy among individuals at low CV disease risk is ongoing [6, 22–28]. Global implementation of the recent ACC/AHA guidelines [5] would considerably increase the number of potential candidates for statin therapy [25]. Our findings indicate that two thirds of the apparently healthy statin initiators did not have such a change in their health status that would have prompted initiation of other CV or diabetes medication within 2 years of initiation. This suggests that a substantial proportion of initiators were at low CV disease risk. Unfortunately, we did not have access to many important risk factors, such as smoking, family history, blood pressure, and lipids, which hampers the assessment of the risk level of our study population and the magnitude of potential statin overuse. However, in a population-based, cross-sectional study of a representative sample of Finnish adult population in 2007 it was estimated that one third of female statin users without established CV disease had a 10-year-risk of future events lower than 5% [29].

Our study has several strengths. Due to the large number of observations we had power to detect differences in model fit across the models. This is crucial because the true number of subpopulations remains unknown. We decided to model accumulation of additional CV or diabetes drugs among initiators of these drugs only as the model for the whole population of statin initiators (GMM with zero-inflated Poisson) was not estimable. Adding covariates to predict the class membership in the chosen model did not essentially change the classification and thus, we did not report the results of those models; instead, we compared the baseline characteristics of the classes via simple statistical tests. Loss to follow-up due to long-term institutionalization or death was negligible in our cohort (1% of the initiators of additional CV or diabetes drugs) and thus we did not need to extend the modeling to allow for missing not at random pattern for missing data. During the time of data collection, all reimbursable drugs were captured by the Prescription Register [30] and the definition of accumulating drugs was kept constant as no novel CV drugs were launched onto the Finnish market in that period. Furthermore, a CV drug purchase in the Prescription Register has been found to be a valid marker of CV disease; when compared with the hospital discharge register, the sensitivity of the prescription register in identifying patients with any CV disease was 99% in 2006 [12].

Our study had some limitations due to reliance on register data. We could not capture the use of low-dose acetylsalicylic acid which is not reimbursed resulting in underestimation of the number of accumulated drugs. Conversely, the number of initiations of antihypertensive drugs may be higher than the number of initiators as a patient may need to test several antihypertensive drugs in order to find the most suitable one. This may have inflated the number of accumulated CV risks reflected by the number of drugs. As mentioned above, we did not have data on all CV disease risk factors that may be detectable in patient records outside the hospital setting to estimate the magnitude of CV risk in the study population. Furthermore, in theory, discontinuation of statin therapy due to adverse events could have resulted in a rapid initiation of CV drugs for the modification of other CV risk factors. Unfortunately, we had no data on adverse events during statin therapy, such as muscle symptoms or increase in hepatic enzyme levels [5].

In conclusion, two thirds of the apparently healthy statin initiators did not use any other CV or diabetes drug during the 2-year follow-up since statin initiation, suggesting that their initial classification as being at low CV risk at the time of initiation was correct. However, one third of the apparently healthy had concurrent drug-modifiable CV risk factors or even existing CV disease at the time of statin initiation or shortly thereafter. In future register-based studies of statin initiators at low CV risk, the definition of low-risk population should exclude statin initiators who initiate other CV or diabetes medication simultaneously with statin in order to avoid misclassification.

## Supporting Information

S1 Table Number of initiations of cardiovascular and diabetes drugs stratified by time since statin initiation among the 4 097 apparently healthy patients initiating statin therapy (a patient can initiate several other drugs). (PDF)

S1 Fig Representation of a quadratic growth mixture model for 5 panel waves (u_i_ = measured count variable). (PDF)
