# Supplementary material for: Accumulation of Cardiovascular and Diabetes Medication among Apparently Healthy Statin Initiators
Source: PLoS One. 2015 Feb 6;10(2):e0117182. doi: 10.1371/journal.pone.0117182 (PMC4319777; doi:10.1371/journal.pone.0117182)
Supplement: S1 Table — (PDF) [file pone.0117182.s001.pdf]

Table S1. Number of initiations of cardiovascular and diabetes drugs stratified by time since statin initiation among the 4 097 apparently healthy patients initiating statin therapy (a patient can initiate several other drugs)

	Days since statin initiation					
	0	1–179	180–364	365–547	548–730	0–730
	n (%)					
Diabetes drugs	247 (11.4)	136 (7.8)	108 (10.9)	130 (13.6)	111 (12.0)	732 (10.8)
Other antithrombotic agents but warfarin	127 (5.8)	169 (9.7)	107 (10.8)	101 (10.6)	92 (9.9)	596 (8.8)
Warfarin	34 (1.6)	40 (2.3)	33 (3.3)	26 (2.7)	33 (3.6)	166 (2.5)
Cardiac glycosides and antiarrhythmics	7 (0.3)	10 (0.6)	13 (1.3)	6 (0.6)	16 (1.7)	52 (0.8)
Organic nitrates	277 (12.7)	163 (9.4)	89 (9.0)	98 (10.3)	83 (9.0)	710 (10.5)
Centrally acting antihypertensive drugs	3 (0.1)	7 (0.4)	4 (0.4)	2 (0.2)	5 (0.5)	21 (0.3)
Diuretics	112 (5.2)	129 (7.4)	89 (9.0)	81 (8.5)	81 (8.8)	492 (7.3)
Beta blockers	444 (20.4)	374 (21.5)	201 (20.4)	163 (17.1)	186 (20.1)	1 368 (20.2)
Selective calcium channel blockers	129 (5.9)	130 (7.5)	90 (9.1)	90 (9.4)	94 (10.2)	533 (7.9)
ACEI or ARB	794 (36.5)	578 (32.3)	253 (25.6)	256 (26.9)	224 (24.2)	2 105 (31.1)
Total	2 174 (100.0)	1 736 (100.0)	987 (100.0)	953 (100.0)	925 (100.0)	6 775 (100.0)

Abbreviations: ACEI, angiotensin-converting enzyme inhibitor; ARB, angiotensin receptor blocker
