# Supplementary material for: Accumulation of Cardiovascular and Diabetes Medication among Apparently Healthy Statin Initiators
Source: PLoS One. 2015 Feb 6;10(2):e0117182. doi: 10.1371/journal.pone.0117182 (PMC4319777; doi:10.1371/journal.pone.0117182)

Figure S1. Representation of a quadratic growth mixture model for 5 panel waves (u_i= measured count variable).

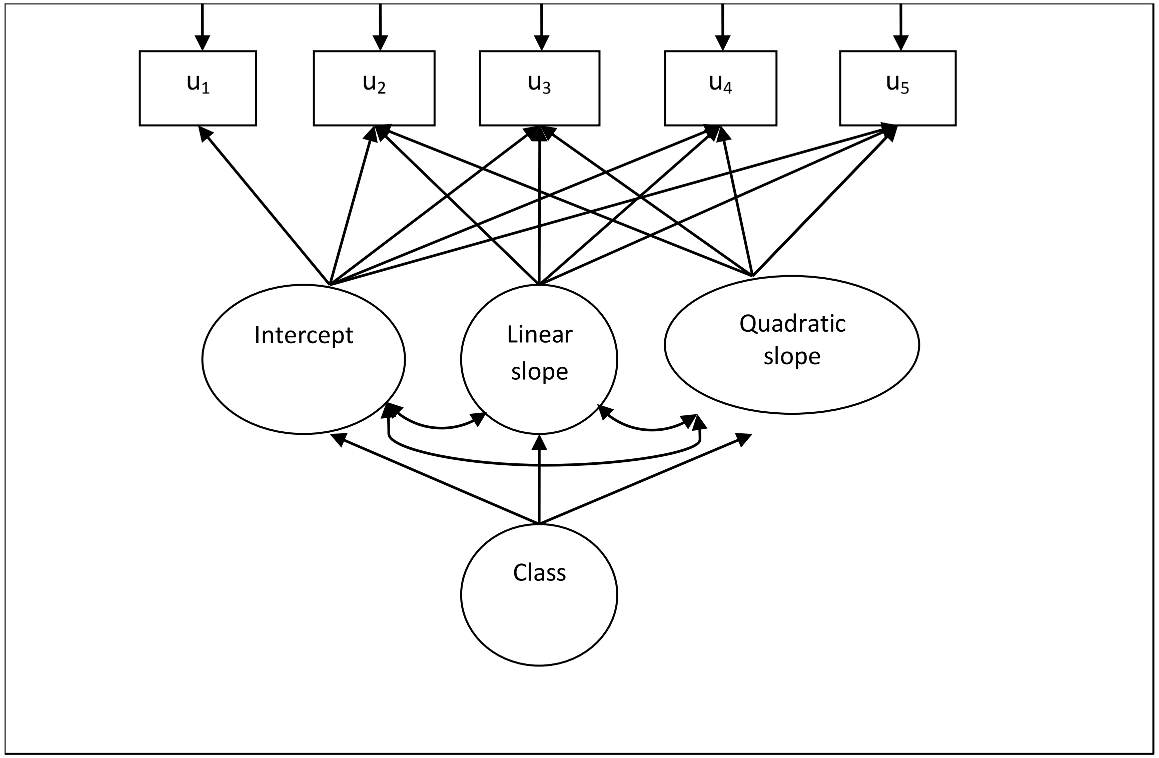

Supplement: S1 Fig — (PDF) [file pone.0117182.s002.pdf]
